# Spontaneous Arterial Hemorrhage of the Hand Resulting in Compartment Syndrome

**Published:** 2015-07-31

**Authors:** Eric Gallagher, Todd Ruiter

**Affiliations:** ^a^Department of Orthopedic Surgery, Western Michigan University Homer Stryker MD School of Medicine, Kalamazoo; ^b^Borgess Medical Center, Kalamazoo, Mich

**Keywords:** hand, compartment syndrome, fasciotomy, measurement, hematoma

**Figure:**
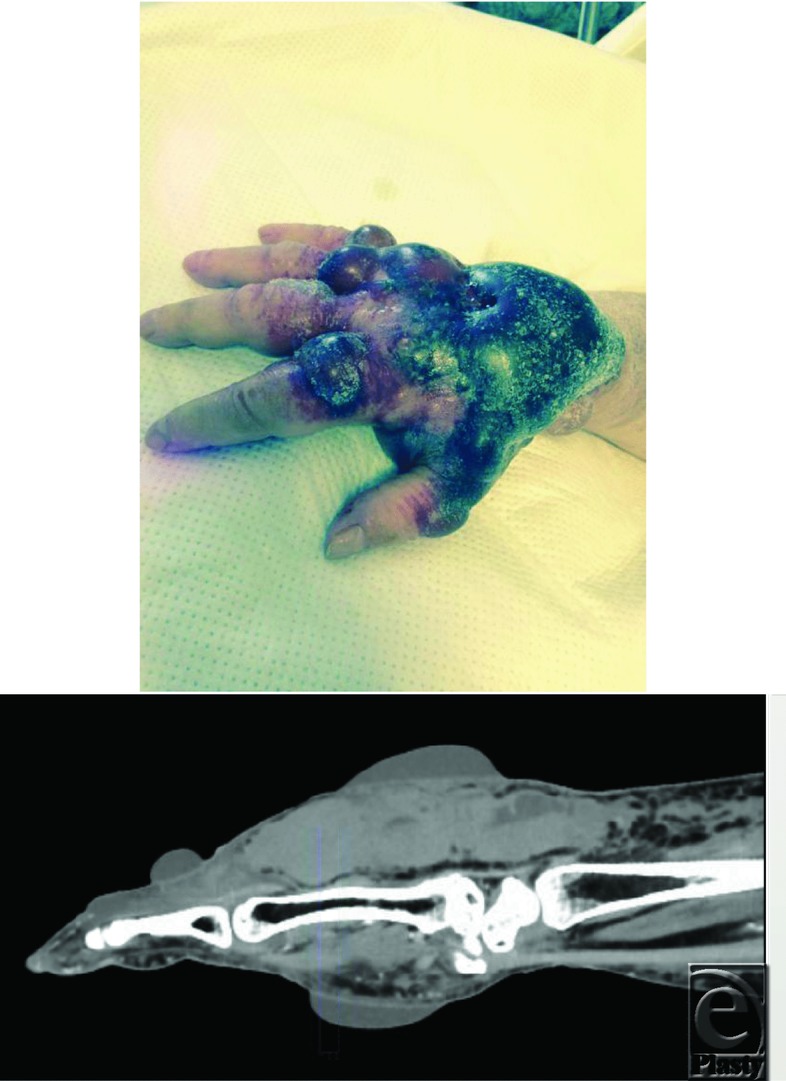


**Figure:**
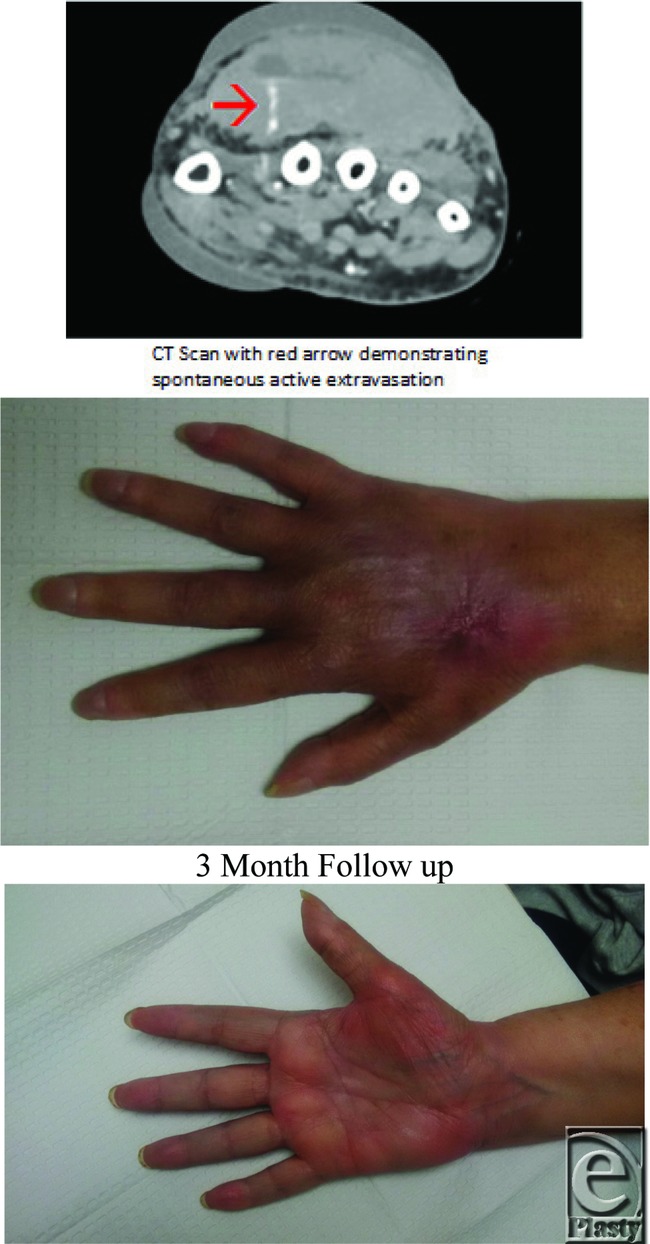


## DESCRIPTION

A 70-year-old woman presented to the emergency department with severe pain and swelling of her hand. A computed tomographic scan demonstrated a large hematoma with active contrast extravasation. Serial examinations noted an expanding hematoma, worsening pain, and evidence of acute compartment syndrome (ACS). Emergent evacuation of the hematoma, limited compartment release with subfascial drain placement, and correction of underlying coagulopathy were pursued.

## QUESTIONS

1. **What are the signs and symptoms of compartment syndrome?**
2. **How do you measure compartment pressures?**
3. **What is the pressure cutoff for determining compartment syndrome?**
4. **How do you release the compartments of the hand?**

## DISCUSSION

Acute compartment syndrome is a serious diagnosis requiring urgent intervention. Most commonly, ACS results from high-energy injuries to the tibia or forearm and occurs within the first 36 to 72 hours after the injury.^1^^,^^2^ Bleeding disorders and anticoagulants are also risk factors.^1^^,^^2^ Early findings are pain with passive muscle stretch and swollen tense compartments.^1^^-^3 Pain out of proportion to injury is a common finding that should prompt high suspicion.^2^^,^^4^ These symptoms progress to sensory deficits and muscle weakness.^1^^-^3 Very late findings are pallor, pulselessness, paralysis, and poikilothermia.^1^ These late findings represent complete ischemia and a poor prognosis.^1^ These clinical findings have a low sensitivity but high specificity.^1^^,^^3^ Our patient did not have a traumatic injury but was anticoagulated on Coumadin (warfarin) for chronic atrial fibrillation, with an initial INR (international normalized ratio) of 5.3. She had severe pain with passive stretch and swollen, tense compartments that were worsening on serial examinations.

The diagnosis of ACS is mostly clinical. However, findings may be inconclusive or the patient may not be reliable due to mental status or confounding injuries. In these situations, it is important to have an accurate and reliable way to measure compartment pressures. Many instruments have been described to measure compartment pressures. The 3 most commonly used instruments are arterial line manometer, handheld Stryker system, and Whiteside manometer.^3^ An analysis of these 3 methods with 3 different types of needles (straight, side port, and slit catheter) was performed to determine the most reliable and reproducible way to determine compartment pressures.^5^ This study showed that side-port needles and slit catheters are more accurate than straight needles.^5^ The arterial line manometer is the most accurate.^5^ The Stryker needle is also very accurate.^5^ The Whiteside manometer may be the least precise instrument.^5^

Sources vary on what pressure constitutes ACS. Some define ACS as a difference between diastolic blood pressure and compartment pressure measuring less than 20 mm Hg.^4^ Others define it as a difference between MAP (mean arterial pressure) and compartment pressure of less than 30 mm Hg.^4^ Others define it as an absolute compartment pressure of less than 30 mm Hg.^4^ These cutoffs should be used as an adjunct to clinical symptoms.

The hand is composed of 10 compartments: 4 dorsal interosseous, three volar interosseous, adductor pollicis, hypothenar muscles, and thenar muscles. To release the compartments of the hand, 4 separate incisions must be made (see the Figure).^1^ Two incisions are required dorsally: one over the second metacarpal and one over the fourth metacarpal.^1^^,^^3^ Through the second metacarpal incision, the first and second dorsal interossei can be released with incisions along both sides of the metacarpal.^1^^,^^6^ Blunt dissection along the radial aspect of the second metacarpal can be used to release the volar interosseous and adductor pollicis.^1^ In the same manner, the third and fourth dorsal interossei may be released through the fourth metacarpal incision.^1^^,^^6^ The remaining volar interosseous compartments are accessed through deep blunt dissection along the radial aspect of the fourth and fifth metacarpals.^1^^,^^6^ The thenar and hypothenar compartments should be released with incisions at the glabrous skin edge on the radial and ulnar aspects of the hand.^1^^,^^6^ If there is any concern for compression in the carpal tunnel and/or Guyon's canal, these should be released.^1^ If there is a concern for finger compartment syndrome, midaxial incisions should be made to release the pressure.^1^^,^^6^ These incisions should be made along the radial side of the thumb and the small finger and ulnar aspect of the remaining digits as depicted.^1^ In our patient, a dorsal incision over the second metacarpal was made and the first dorsal interosseous, second dorsal interosseous, volar interosseous, and adductor pollicis compartments were released. This provided complete evacuation of the hematoma and relief of her ACS.

Compartment syndrome is a surgical emergency that may result in severe, limb-threatening consequences if left untreated. Presenting signs and symptoms guide diagnosis, but often invasive pressure measuring is necessary. Our patient had an excellent outcome due to timely diagnosis and urgent release. At 3-month follow-up, she had returned to her normal activities of daily living without any perceived disability and was able to demonstrate full range of motion, symmetric grip strength, and function of intrinsic musculature.

## Figures and Tables

**Figure:**
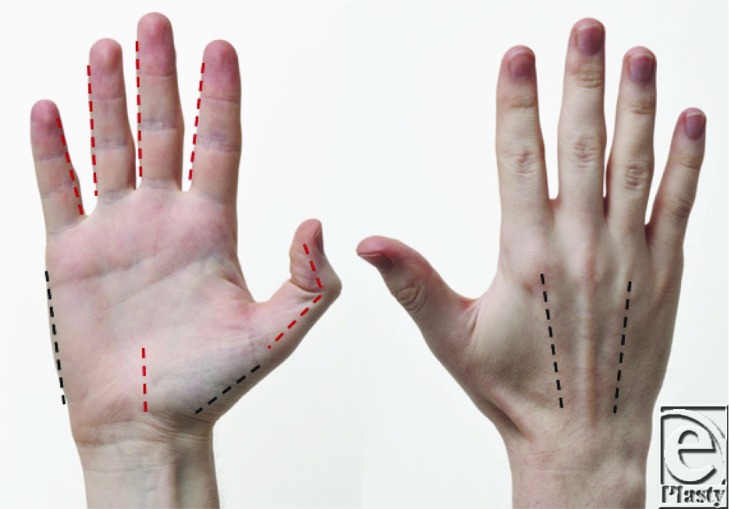
Fasciotomy incisions in black and optional carpal tunnel and midaxial incisions in red.
